# Supplementary material for: Trends of cleft surgeries and predictors of late primary surgery among children with cleft lip and palate at the University College Hospital, Nigeria: A retrospective cohort study
Source: PLoS One. 2023 Jan 3;18(1):e0274657. doi: 10.1371/journal.pone.0274657 (PMC9810161; doi:10.1371/journal.pone.0274657)

PLOS ONE

Trends of cleft surgeries and predictors of late primary surgery among children with cleft lip and palate at the University College Hospital, Nigeria: a retrospective cohort study

--Manuscript Draft--

**Manuscript Number:**	PONE-D-22-24340
**Article Type:**	Research Article
**Full Title:**	Trends of cleft surgeries and predictors of late primary surgery among children with cleft lip and palate at the University College Hospital, Nigeria: a retrospective cohort study
**Short Title:**	Trends of cleft surgeries and predictors of late primary cleft surgery
**Corresponding Author:**	Afieharo Igbibia Michael<br>University of Ibadan<br>Ibadan, Oyo NIGERIA
**Keywords:**	cleft lip; cleft palate; cleft surgery; late primary cleft surgery
**Abstract:**	Background:<br>Cleft of the lip and palate is the most common craniofacial birth defect with a worldwide incidence of one in 700 live births. Early surgical repairs are aimed at improving appearance, speech, hearing, psychosocial development and avoiding impediments to social integration. Many interventions including the Smile Train partner model have been introduced to identify and perform prompt surgical procedures for the affected babies. However, little is known about the trends of the incidence and surgical procedures performed at our hospital and the relationship between the clinical characteristics of the patients and the timing of primary repairs.<br>Objective<br>To determine the trends in cleft surgeries, patterns of cleft surgeries and identify factors related to late primary repair at the University College Hospital, UCH, Ibadan, Nigeria.<br>Methods<br>A retrospective cohort study and trends analysis of babies managed for cleft lip and palate from January 2007 to January 2019 at the UCH, Ibadan was conducted. The demographic and clinical characteristics were extracted from the Smile Train enabled cleft database of the hospital. The annual trends in rate of cleft surgeries (number of cleft surgeries per 100,000 live births) was represented graphically. Chi square test and Student's t-test were utilised to assess the association between categorical and continuous variables and delay in cleft surgery (≥12 months for lip repair, ≥18months for palatal repair). Kaplan-Meier graphs with log-rank test was used to examine the association between sociodemographic variables and the outcome (late surgery). Univariable and multivariable Cox proportional hazard regression was conducted to obtain the hazard or predictors of delayed cleft lip surgery. Stata version 17 (Statacorp, USA) statistical software was utilised for analysis<br>Results<br>There were 314 cleft surgeries performed over the thirteen-year period of study. The male to female ratio was 1.2:1. The mean age of the patients was 58.08 ± 99.65 months. The median age and weight of the patients were 11 (IQR:5-65) months and 8 (IQR: 5.5-16) kg respectively. Over half (n=184, 58.6 %) of the cleft surgeries were for primary repairs of the lip and a third (n=94, 29.9%) were surgeries for primary repairs of the palate. Millard's rotation advancement flap was the commonest lip repair technique with Fishers repair introduced within two years into the end of the study. Bardachs two flap palatoplasty has replaced Von Lagenbeck palatoplasty as the commonest method of palatal repair. The prevalence of late primary cleft lip repair was about a third of the patients having primary cleft lip surgery while the prevalence of late palatal repair was more than two thirds of those who received primary palatoplasty. Compared with children who had bilateral cleft lip, children with unilateral cleft lip had a significantly increased risk of late primary repair (Adj HR: 22.4, 955 CI: 2.59 - 193.70, P-value = 0.005).

Powered by Editorial Manager® and ProduXion Manager® from Aries Systems Corporation

	Conclusion<br>There has been a change from Von Lagenbeck palatoplasty to Bardachs two-flap palatoplasty. Intra-velar veloplasty and Fisher's method of lip repair were introduced in later years. There was a higher risk of late primary repair in children with unilateral cleft lip.
Order of Authors:	Afieharo Igbibia Michael
	Gbenga Olorunfemi
	Adeola Olusanya
	Odunayo Oluwatosin
Additional Information:	
**Question**	**Response**
**Financial Disclosure**<br><br>Enter a financial disclosure statement that describes the sources of funding for the work included in this submission. Review the submission guidelines for detailed requirements. View published research articles from *PLOS ONE* for specific examples.<br><br>This statement is required for submission and **will appear in the published article** if the submission is accepted. Please make sure it is accurate.<br><br>**Unfunded studies**<br>Enter: *The author(s) received no specific funding for this work.*<br><br>**Funded studies**<br>Enter a statement with the following details:<br>• Initials of the authors who received each award<br>• Grant numbers awarded to each author<br>• The full name of each funder<br>• URL of each funder website<br>• Did the sponsors or funders play any role in the study design, data collection and analysis, decision to publish, or preparation of the manuscript?<br>• **NO** - Include this sentence at the end of your statement: *The funders had no role in study design, data collection and analysis, decision to publish, or preparation of the manuscript.*<br>• **YES** - Specify the role(s) played.<br><br>* typeset	The author(s) received no specific funding for this work.
**Competing Interests**	The authors have declared that no competing interests exist.

Powered by Editorial Manager® and ProduXion Manager® from Aries Systems Corporation

Use the instructions below to enter a competing interest statement for this submission. On behalf of all authors, disclose any competing interests that could be perceived to bias this work—acknowledging all financial support and any other relevant financial or non-financial competing interests.<br><br>This statement is required for submission and **will appear in the published article** if the submission is accepted. Please make sure it is accurate and that any funding sources listed in your Funding Information later in the submission form are also declared in your Financial Disclosure statement.<br><br>View published research articles from *PLOS ONE* for specific examples.<br><br>**NO authors have competing interests**<br><br>Enter: *The authors have declared that no competing interests exist.*<br><br>**Authors with competing interests**<br><br>Enter competing interest details beginning with this statement:<br><br>*I have read the journal's policy and the authors of this manuscript have the following competing interests: [insert competing interests here]*<br><br>* typeset	
**Ethics Statement**<br><br>Enter an ethics statement for this submission. This statement is required if the study involved:<br><br>• Human participants<br>• Human specimens or tissue<br>• Vertebrate animals or cephalopods<br>• Vertebrate embryos or tissues	The study was approved by the University of Ibadan/University College Hospital institutional review board. (Ethics review number: NHREC/05/01/2008a)

• Field research<br><br>Write "N/A" if the submission does not require an ethics statement.<br><br>General guidance is provided below. Consult the submission guidelines for detailed instructions. **Make sure that all information entered here is included in the Methods section of the manuscript.**	

**Format for specific study types**<br><br>**Human Subject Research (involving human participants and/or tissue)**<br>• Give the name of the institutional review board or ethics committee that approved the study<br>• Include the approval number and/or a statement indicating approval of this research<br>• Indicate the form of consent obtained (written/oral) or the reason that consent was not obtained (e.g. the data were analyzed anonymously)<br><br>**Animal Research (involving vertebrate animals, embryos or tissues)**<br>• Provide the name of the Institutional Animal Care and Use Committee (IACUC) or other relevant ethics board that reviewed the study protocol, and indicate whether they approved this research or granted a formal waiver of ethical approval<br>• Include an approval number if one was obtained<br>• If the study involved *non-human primates*, add *additional details* about animal welfare and steps taken to ameliorate suffering<br>• If anesthesia, euthanasia, or any kind of animal sacrifice is part of the study, include briefly which substances and/or methods were applied<br><br>**Field Research**<br><br>Include the following details if this study involves the collection of plant, animal, or other materials from a natural setting:<br>• Field permit number<br>• Name of the institution or relevant body that granted permission	
**Data Availability**<br><br>Authors are required to make all data underlying the findings described fully available, without restriction, and from the time of publication. PLOS allows rare exceptions to address legal and ethical concerns. See the PLOS Data Policy and FAQ for detailed information.	No - some restrictions will apply

A Data Availability Statement describing where the data can be found is required at submission. Your answers to this question constitute the Data Availability Statement and **will be published in the article**, if accepted.<br><br>**Important:** Stating 'data available on request from the author' is not sufficient. If your data are only available upon request, select 'No' for the first question and explain your exceptional situation in the text box.<br><br>Do the authors confirm that all data underlying the findings described in their manuscript are fully available without restriction?	
**Describe where the data may be found in full sentences. If you are copying our sample text, replace any instances of XXX with the appropriate details.**<br><br>• If the data are **held or will be held in a public repository**, include URLs, accession numbers or DOIs. If this information will only be available after acceptance, indicate this by ticking the box below. For example: *All XXX files are available from the XXX database (accession number(s) XXX, XXX.*).<br>• If the data are all contained **within the manuscript and/or Supporting Information files**, enter the following: *All relevant data are within the manuscript and its Supporting Information files.*<br>• If neither of these applies but you are able to provide **details of access elsewhere**, with or without limitations, please do so. For example:<br><br>*Data cannot be shared publicly because of [XXX]. Data are available from the XXX Institutional Data Access / Ethics Committee (contact via XXX) for researchers who meet the criteria for access to confidential data.*<br><br>*The data underlying the results presented in the study are available from (include the name of the third party*	The data underlying the results presented in the study are available from Smile Train Express

Powered by Editorial Manager® and ProduXion Manager® from Aries Systems Corporation

and contact information or URL).<br>• This text is appropriate if the data are owned by a third party and authors do not have permission to share the data.<br><br>* typeset	
Additional data availability information:	Tick here if your circumstances are not covered by the questions above and you need the journal's help to make your data available.

Powered by Editorial Manager® and ProduXion Manager® from Aries Systems Corporation

Manuscript Click here to access/download;Manuscript;Plos One Aug 22.docx

Trends of cleft surgeries and predictors of late primary surgery among children with cleft lip and palate at the University College Hospital, Nigeria: a retrospective cohort study

Afieharo I. Michael[1,2*], Gbenga Olorunfemi[3,4], Adeola A. Olusanya[5], Odunayo M. Oluwatosin[1,2]

[1]Department of Surgery, College of Medicine. University of Ibadan, Ibadan, Nigeria

[2]Department of Plastic, Reconstructive and Aesthetic Surgery, University College Hospital, Ibadan, Nigeria

[3]Department of Obstetrics & Gynaecology, Lagos University Teaching Hospital, Lagos, Nigeria,

[4]Division of Epidemiology and Biostatistics, School of Public Health, University of Witwatersrand, Johannesburg, South Africa

[5]Department of Oral and Maxillofacial Surgery, Faculty of Dentistry. University of Ibadan, Ibadan, Nigeria

[*]Corresponding author

afiemichael@gmail.com(AIM)

Abstract

Background:

Cleft of the lip and palate is the most common craniofacial birth defect with a worldwide incidence of one in 700 live births. Early surgical repairs are aimed at improving appearance, speech, hearing, psychosocial development and avoiding impediments to social integration. Many interventions including the Smile Train partner model have been introduced to identify and perform prompt surgical procedures for the affected babies. However, little is known about the trends of the incidence and surgical procedures performed at our hospital. Nothing is also known about the relationship between the clinical characteristics of the patients and the timing of primary repairs

Objective

To determine the trends in cleft surgeries, patterns of cleft surgeries and identify factors related to late primary repair at the University College Hospital, UCH, Ibadan, Nigeria.

Methods

A retrospective cohort study and trends analysis of babies managed for cleft lip and palate from January 2007 to January 2019 at the UCH, Ibadan was conducted. The demographic and clinical characteristics were extracted from the Smile Train enabled cleft database of the hospital. The annual trends in rate of cleft surgeries (number of cleft surgeries per 100,000 live births) was represented graphically. Chi square test, Student's t-test and Mann Whitney U were utilised to assess the association between categorical and continuous variables and delay in cleft surgery (≥12 months for lip repair, ≥18months for palatal repair). Kaplan-Meier graphs with log-rank test was used to examine the association between sociodemographic variables and the outcome (late

surgery). Univariable and multivariable Cox proportional hazard regression was conducted to obtain the hazard or predictors of delayed cleft lip surgery. Stata version 17 (Statacorp, USA) statistical software was utilised for analysis

Results

There were 314 cleft surgeries performed over the thirteen-year period of study. The male to female ratio was 1.2:1. The mean age of the patients was 58.08 ± 99.65 months. The median age and weight of the patients were 11 (IQR:5-65) months and 8 (IQR: 5.5-16) kg respectively. Over half (n=184, 58.6 %) of the cleft surgeries were for primary repairs of the lip and a third (n=94, 29.9%) were surgeries for primary repairs of the palate. Millard's rotation advancement flap was the commonest lip repair technique with Fishers repair introduced within two years into the end of the study. Bardachs two flap palatoplasty has replaced Von Lagenbeck palatoplasty as the commonest method of palatal repair. The prevalence of late primary cleft lip repair was about a third of the patients having primary cleft lip surgery while the prevalence of late palatal repair was more than two thirds of those who received primary palatoplasty. Compared with children who had bilateral cleft lip, children with unilateral cleft lip had a significantly increased risk of late primary repair (Adj HR: 22.4, 955 CI: 2.59 - 193.70, P-value = 0.005).

Conclusion

There has been a change from Von Lagenbeck palatoplasty to Bardachs two-flap palatoplasty. Intra-velar veloplasty and Fisher’s method of lip repair were introduced in later years. There was a higher risk of late primary repair in children with unilateral cleft lip.

Introduction

Cleft of the lip and palate is the most common craniofacial birth defect with a worldwide incidence of one in 700 live births (1). This burden is higher in low- and middle-income countries with a higher number of untreated clefts (2). Early surgical repairs are aimed at improving appearance, speech, hearing, psychosocial development and avoiding impediments to social integration (3). Some guidelines have been put forth to optimize safety, ensure adequacy of repair, and improve function while limiting morbidity to the patient with the cleft deformity(4,5). Surgical procedures should be well planned to reduce exposure to anaesthesia from multiple surgeries (3).

One of the common guidelines for the timing of surgical cleft lip repair is the rule of ten (4). Although its validity is questioned, the "rule of ten" guideline is still widely used(6–8). By this rule, children who are ten weeks old, attained the weight of five kilograms and have a haemoglobin of 10g/dl can commence primary lip repair if there are no other mitigating medical conditions (9). This rule is also employed in our institution. The American Cleft Palate-Craniofacial Association (ACPA) introduced parameters to evaluate and treat patients with cleft lip and palate (3). The earlier edition of ACPA guidelines recommended that lip repair be completed by six months and palatal repair by 18 months while the latest revision provides that initiation of lip repair be commenced before 12 months and palatal repair completed by 18 months (3). The association further advised that earlier surgeries are desired if the conditions are right.

Based on the ACPA guidelines, Cassell et al. from a resourced nation, reported nearly 90% of the patients in their study had had their surgeries by the age of six months (10). A study by

Cornway et al. looked at cleft surgeries across Africa. They reported a mean age of nine years for commencement of primary surgeries (11). From China an average age of 1.8 years for lip repair and 5.9 years for palatal repair was reported by Kling et al (12). A study from Northern Nigeria reported a mean age of 12.4 years for the commencement of primary surgeries in their cohort of 149 patients with cleft anomalies (6). Onah et al also reported a delay in commencement of primary surgeries for cleft of the palate in more than half of their study population in southeast Nigeria (13).

The Smile Train, the world's largest cleft centred charity-based organisation that provides funding for children with clefts began partnership with Nigeria in 2002 aimed at prompt and safe cleft surgery (14). Nevertheless, some reports of delay in cleft repair still occurred in the country. (6,13,15,16). The demographic and clinical parameters that may be responsible for delay in primary surgeries have not been fully explored in Nigeria. This lack of information makes it difficult to design innovative methods to mitigate this problem, help the patients attain early repairs and improve their quality of life. A critical review cleft surgical trends and delay patterns in our hospital with an understanding of the demographic and clinical predictors of this delay would inform strategies at curbing these occurrences. Hence, we aimed to know the trends in cleft surgeries, determine the patterns of cleft surgeries and identify factors related to late primary repair.

Methods

Study design and setting

This was a retrospective cohort study of all cleft surgeries from January 2007 to December 2019. The study was carried out at the University College Hospital, Ibadan, a tertiary care, federal government owned, facility in South-Western Nigeria. The hospital commenced partnership with the Smile Train in 2007 and has been providing free cleft care to patients with clefts. The rule of 10 as proposed by Wilhelmsen and Musgrave [4] and modified by Millard [8] is used for guiding scheduling of our patients for primary lip repair. The data of patients who received cleft care are entered into the Smile Train Express (STX). This database is specific to the institution. All patients who received care gave informed consent for surgery and for entry into and utilization of their details in this database. Anonymised data was extracted from the STX database and crosschecked with existing patient's case record forms and case notes. Data on annual live births were obtained from the hospital records department. The study was approved by the institutional review board. (Ethics review number: NHREC/05/01/2008a)

Definition of terms

Typical cleft deformities were classified according to the ACPA classification of clefts into clefts of the primary palate and cleft of the secondary palate. Further sub classifications were done in line with the International Classification of Diseases (5)(ICD) 10 classification into Cleft Lip (CL), which is cleft of the primary palate alone, Cleft lip and palate (CLP), these are clefts that involve both the primary and secondary palates and Cleft palate (CP), which is an isolated cleft

of the secondary palate. Further subdivisions used in this study were based on severity of the defect (incomplete/complete), laterality (right/left/bilateral).

Atypical clefts were clefts that involved any other part of the face that differed from the commoner pattern of cleft lip and palate. Here they were classified according to Paul Tessier(17).

Primary surgeries were defined as the first surgeries performed on any anatomical region of the cleft.

Secondary surgeries - Subsequent surgeries performed after the primary surgery on the same anatomical region of the cleft.

Variables

Primary outcome measure was Late primary surgery (age at surgery ≥ 12 months for lip repair and ≥ 18 months for palatal repair). Independent variables were age, gender, weight, type of deformity, severity of the deformity, age at surgery and year of surgery. Only primary surgeries of typical clefts were utilised for bivariate and multivariable a analysis to determine the primary outcomes, as such atypical clefts and secondary surgeries were not analyzed further.

Data analysis

Categorical variables are presented as frequency and percentages while continuous variables are presented as mean and standard deviation where normally distributed or median and interquartile range if not normally distributed. Yearly trends in rate of cleft surgeries (number of cleft surgeries per 100,000 live births) are presented in graphical form. Chi square test was used to assess the association between sociodemographic characteristics and delay in cleft surgery. Student's t-test and Mann Whitney U were utilised to assess the association between continuous variables and

delay in cleft surgery (≥12 months for lip repair, ≥18months for palatal repair). Pearson's correlation coefficient was utilised to determine the association between weight and age at surgery. The time-variate variable was "time to cleft lip surgery". Kaplan-Meier graphs with log-rank test was used to examine the association between sociodemographic variables and the outcome (late surgery). Univariable and multivariable Cox proportional hazard regression was conducted to obtain the hazard or predictors of delayed cleft lip surgery. P-value of ≤ 0.05 was considered as statistically significant. Stata version 17 (Statacorp, USA) statistical software was utilised for analysis.

Results

Demographic and clinical characteristics

There were 314 cleft surgeries performed over the thirteen-year period of study (January 2007-December 2019). Table 1 shows the demographic and clinical characteristics of the patients. The male to female ratio was 1.2:1. The mean age of the patients was 58.08 ± 99.65 months. The median age and weight of the patients were 11 (IQR:5-65) months and 8 (IQR: 5.5-16) kg respectively. About half (n=167, 51.3%) of the patients were infants (aged ≤ 12months) while one-tenth (10.2%, n = 32) of the operated patients were adults. Nearly half of the patients had a primary diagnosis of cleft lip (n=130/314, 41.4%) and about one-third had cleft of the lip and palate (n=119/314, 37.9%). Unilateral defects were almost twice as common on the left 99 (63.9%) as on the right 56 (36.1%). Of the 314 performed cleft surgeries, over half (n=184, 58.6 %) were for primary repairs of the lip and a third (n=94, 29.9%) were surgeries for primary repairs of the palate. Isolated defects of the palate were 50 (53.2%). Within this group was a female predominance of 30 (66.7%). The distribution of primary surgeries between incomplete cleft lip, complete cleft lip and complete cleft lip and palate were fairly even. Primary surgeries for complete cleft palate were more than twice the number for incomplete cleft palate.

Table 1: Demographic and clinical characteristics of the patients

**Variable**	**Frequency**<br><br>**N (%)**
**Gender (N=314)**	
Female	173(44.9)
Male	141 (55.1)
**Age (Median, IQR), months**	11(5-65)
**Age category (N=314)**	
Infants	161(51.3)
Toddler	43(13.7)
Pre school	14(4.5)
School age	42(13.4)
Adolescent	22(7.0)
Adult	32(8.9)
**Weight (Median, IQR), kg**	8.0(5.5-16)

< 5	34(12.2)
≥5	244(87.8)
**Primary Diagnosis (N=314)**	
Cleft lip	130 (41.4)
Cleft lip and palate	119(37.9)
Cleft palate	53(16.9)
Atypical cleft	12(3.8)
**Type of Surgeries done (N=314)**	
Primary unilateral cleft lip	155 (49.4)
Primary bilateral cleft lip	29 (9.2)
Primary cleft palate	94(29.9)
Primary Atypical cleft	12 (3.8)
Secondary cleft surgery	24 (7.6)
**Severity of the unilateral cleft lip (N=155)**	
Incomplete cleft lip	55 (35.6)
Complete cleft lip	57 (36.7)

Complete cleft lip and palate	43 (27.7)
**Severity of the cleft palate (N=94)**	
Incomplete	24(25.5)
Complete	70 (74.5)

Trends in cleft surgeries

There was a steady rise in the number of cleft surgeries from inception in 2007 with 19 cleft surgeries to a peak of 39 cleft surgeries in 2010 and 2011, fig 1. A steep drop to no cleft surgeries was seen in 2013. A subsequent steady increase was then seen thereafter. However, the number of surgeries has not reached the initial peak. The cleft lip and palate rate per 1000 live births demonstrates three peaks, in the years 2010, 2014 and 2018 with rates of 20.2/1000, 7.1/1000 and 17.4/1000 respectively, fig 2, table 2.

Table 2. Trends in the annual number of clefts and incidence (2007 – 2019)

**Year**	**Total**	**Livebirth**	**Cleft lip**	**Cleft lip rate/1000 live births**	**Cleft palate**	**Cleft palate rate/1000 live births**
2007	19	1752	11	6.3	6	3.4
2008	26	2414	12	5.0	11	4.6

2009	25	2259	18	8.0	5	2.2
2010	39	1928	22	11.4	13	6.7
2011	39	2251	22	9.8	9	4.0
2012	33	2429	19	7.8	8	3.3
2013	0	2,077	0	0.0	0	0.0
2014	29	1,699	20	11.8	9	5.3
2015	7	1,165	4	3.4	3	2.6
2016	17	1,988	14	7.0	2	1.0
2017	23	2,074	11	5.3	8	3.9
2018	27	1,552	15	9.7	10	6.4
2019	30	2,206	16	7.3	10	4.5

Fig 1: Annual trends in cleft surgeries

Fig 2: Annual incidence of cleft surgeries per 1000 live births

Trends in methods of cleft lip and palate repair

The most common method of cleft lip repair was the variant of Millard's rotation advancement flap, fig 3. While the classical Millard's repair was used at the outset in 2007 it's use was discontinued in 2016. The least favoured method of cleft lip repair was the rose Thompson straight line repair. The Fisher repair was introduced in 2017. Between 2007 and 2013 the Von Lagenbeck palatoplasty technique was the most favoured method of palatal repair. This was subsequently taken over by the Bardach's two-flap palatoplasty technique. Intravelar veloplasty and Furlow's palatoplasty techniques were introduced in the year 2014, fig 4.

Fig 3: Annual methods of cleft lip repair

Fig 4: Annual methods of palatal repair

Association of demographic and clinical parameters with age at primary surgery

Nearly all the primary surgeries that were done in children less than 6 months of age were for cleft lip repair as compared with cleft palate repair. Most of the surgeries for cleft palate repair were done in children greater than 18months of age (n=63, 67%). Based on the severity of the

cleft anomaly, children with less severe deformity of the unilateral cleft lip (incomplete cleft) (n=23,56.1%) had their surgeries later (>18months) than children with the more severe anomaly (complete cleft lip and palate) (n=3,7.3%), p=0.001. Similarly, children with unilateral defects had later surgeries compared with those with bilateral defects (4 (97.6%) Vs 1(2,4%)), p=0.002. More children with incomplete palatal defects (n=43, 68%) had later surgeries (>18 months) than children with complete defects (n=20, 31.8%), p=0.05. There was no association between gender and age at primary cleft repair. The association between age at primary surgery and the demographic and clinical parameters are shown in table 3.

Table 3: Association of demographic and clinical parameters with age at primary surgery

**Characteristics**	**Age at Surgery (months)**					**P-value**
	**< 6**<br>**N (%)**	**6 - 11**<br>**N (%)**	**12 – 17**<br>**N (%)**	**≥18**<br>**N (%)**	**Total**<br>**N (%)**	
Weight (Median, IQR), kg	5.4 (5.0-6.0)	7 (5.6 – 8.4)	8.2 (6.5 - 9.0)	23 (13-48)	8.0(5.5-16)	0.0001$
**Gender**						
Male	65(55.6)	25(58.1)	14(58.3)	69(53.1)	173 (55.1)	0.922£

Female	52(44.4)	18 (41.9)	10(41.7)	61(46.9)	141 (44.9)	
**Type of repair**						
Primary	116 (99.2	41 (95.4)	23 (95.8)	110 (84.6)	290 (92.4)	<0.001[Fi]
Secondary	1 (0.9	2 (4.7)	1 (4.2)	20 (15.4)	24 (7.6)	
**Type of primary repair**						
Cleft lip	111 (60.3)	23 (12.5)	8 (4.4)	42 (22.8)	184 (100.0)	<0.001
Cleft palate	1 (1.1)	16 (17.0)	14 (14.9)	63 (67.1)	94 (100.0)	
**Severity of unilateral Cleft**						
Incomplete cleft lip	24 (26.3)	7 (46.7)	1 (12.5)	23 (56.1)	55 (34.5)	0.001[Fi]
Complete cleft lip	34 (37.4)	3 (20.0)	5 (62.5)	15 (36.6)	57 (36.8)	

Complete cleft lip and palate	33 (36.3)	5 (33.3)	2 (25.0)	3 (7.3)	43 (27.7)	
**Severity of cleft palate**						
Incomplete cleft palate	0 (0.0)	14 (87.5)	13 (92.9)	43 (68.3)	70 (74.5)	0.05[Fi]
Complete cleft palate	1 (100.0)	2 (12.5)	1 (7.1)	20 (31.8)	24 (25.5)	
**Laterality of cleft lip**						
Unilateral	91 (82.0)	15 (65.2)	8 (100.0)	41 (97.6)	155 (84.2)	0.002[Fi]
Bilateral	20 (18.0)	8 (34.8)	0 (0.0)	1 (2.4)	29 (15.8)	

[$:] Kruskal Wallis; [£] Pearson’s Ch-square; [Fi]Fisher’s exact

Kaplan Meir plot of the time from birth to primary surgery

The total time of follow-up was 14,770 child-month and the rate of primary cleft surgery was 18 per thousand child-months after birth. Furthermore, the median time from birth to primary surgery was 10months while 25% of the babies had surgery after 48 months. (Fig 5A, Table 4)

Figure 5A. Kaplan Meir plot of the time to cleft surgeries

Table 4. Association between survival time and gender and type of primary surgery

**Characteristics**	**Follow-up time (months)**	**Rate of surgery (per thousand child-month)**	**Number of babies**	**Survival time (months)**			**P-value£**
				**25%**	**50%**	**75%**	
**Overall**	14,770	18.8	278	4	10	48	
**Gender**							
Male	8,339	18.6	155	4	10	39	0.90
Female	6,431	19.1	123	5	10	58	
**Weight (Median, IQR), kg**							
< 5	235	144.7	34	3	4	10	
≥5	14,535	16.8	244	5	11	63	
**Type of cleft**							
Unilateral Cleft lip	7,691	20.2	155	3	5	19	< 0.001

Bilateral cleft lip	553	52.4	29	4	5	7	
Cleft lip and palate	6,526	14.4	94	15	30	76	
£ : Logrank test							

There was no statistically significant difference in the survival pattern of time to surgery by gender (logrank P-value = 0.90). Fig 5B, Table 4.

Fig 5B. Kaplan Meir plot of the time to cleft surgeries stratified by gender

However, the rate of primary surgery was least among babies with cleft lip and palate (14.4 per thousand child-months) followed by the rate of primary surgery among babies with cleft lip (20.2 per thousand child-months) and babies with bilateral cleft lip had the highest rate of primary surgery (52.4 per thousand child-months). Furthermore, the median time to surgery was 5 months for both unilateral and bilateral cleft lip surgeries while the median time from birth to primary surgery among babies with both cleft lip and palate was 15 months. Fig 5C.

Fig 5C. Kaplan Meir plot of the time to cleft surgeries stratified by type of cleft defect

Prevalence and predictors of late surgery

Based on the definition of late primary surgery (12 months and beyond for lip repair and 18 months for palate repair) Of the 278 babies that had primary repair, the prevalence of late primary repair

was 40.7% (95% CI: 35.0% - 46.6%), n= 113/278. From Table 5, there was a statistically significant relationship between the type of cleft and hazard of delay in primary repair. Thus, there was a 22 -fold hazard of late primary cleft repair among babies with unilateral cleft lip as compared to the hazard of late repair among babies with bilateral cleft lip (Adj HR: 22.4, 955 CI: 2.59 - 193.70, P-value = 0.005). Likewise, there was a 23 -fold hazard of late primary cleft repair among babies with cleft lip and palate as compared to the hazard of late repair among babies with bilateral cleft lip (Adj HR: 23.4, 95%CI: 2.69 – 202.5, P-value = 0.004).

Table 5. Cox proportional regression of the hazard of late primary repair among babies with cleft lip and palate

**Variable**	**Univariable**			**Multivariable**		**P-value**
	**Hazard ratio**	**95% Confidence interval**	**P-value**	**Hazard ratio**	**95% Confidence interval**	
**Type of cleft**						
Bilateral cleft lip	1.00	Reference	Reference	1.00	Reference	Reference
Unilateral Cleft lip	2.83	0.39 - 20.81	0.31	22.4	2.59 - 193.70	0.005

Cleft lip and palate	4.04	0.55 – 29.54	0.17	23.35	2.69 - 202.54	0.004
**Gender**						
Male	1.00	Reference	Reference	0.95	0.63 - 1.42	0.805
Female	1.08	0.74 - 1.59	0.42			
**Weight (kg)**	0.86	0.84 - 0.89	< 0.001	0.85	0.82 - 0.88	< 0.001
< 5	1.00	Reference	Reference			
≥5	0.10	0.04 - 0.28	< 0.001			

Discussion

We set out to determine the surgical trends and pattern of cleft surgeries at UCH, Ibadan from 2007 – 2019 and evaluated the predictors of late primary cleft surgeries. The period of study covered the commencement of the partnership between smile train international and UCH in offering free cleft care to patients. We found a steep rise in the number of surgeries performed from the onset of the partnership attaining a peak about four years afterwards. Nineteen surgeries were performed in 2007 and it increased to a peak of 39 surgeries in 2010. However, there was fluctuation in the annual number of surgeries, but the peak of 2010 was not attained through the

subsequent years. This was also mirrored by the incidence of cleft surgeries per 1000 live births. Primary cleft lip repairs were the predominant surgeries performed. Modifications of the Millard rotation advancement flap was the most favoured method of lip repair through the years. For palatoplasty, the Von Lagenbecks method was favoured in the earlier 7 years but discontinued and replaced with the Bardach's two-flap palatoplasty in the last 6 years of the study. The less severe anomalies such as incomplete cleft lip, incomplete cleft palate or unilateral defects were significantly associated with late primary repairs. About four out of ten babies in our cohort had late surgeries.

The gender distribution in this study is similar to other studies that have reported an overall male preponderance with female preponderance in Isolated cleft palate. (11,18,19) A study from China however observed more males in patients with isolated cleft palate, which they adduced to likely, cultural female discrimination in their setting.(12) laterality of the anomalies in our study shows a predominance of left sided clefts as reported in other studies.(18,19)

The steep rise in the number of surgeries performed from the onset of the partnership could be because of the increasing awareness of free surgeries, possibly reflecting a mop-up of backlogs of unrepaired clefts. This annual low volume of cleft surgeries has earlier been reported by Olasoji et al. They identified low volume operators in Nigeria that performed not more than ten cleft surgeries in a year. (20) . We found that the trend in secondary cleft surgeries were fairly stable although the prevalence was low. This trend is secondary surgeries is contrary to the observation by Purnell et al. they found that the longer (above 6 years) the partnership of hospitals with Smile Train, the higher were the number of secondary surgeries.(21)

Primary cleft lip surgeries were the predominant cleft surgeries carried out at our hospital similar to reports from other studies in Nigeria and other low- and middle-income countries.(6,22,23) Of these, surgeries on patients with CL were more than those on patients with CLP. However other reviews have reported a preponderance of surgeries on patients with CLP.(11,19) Primary palatoplasties formed a third of the total cleft surgeries in our series. This was quite similar to the series reported by Olutayo et al from a similar tertiary health facility in southwest Nigeria. We found that few proportion of cleft repairs among our cohort were secondary surgeries. In contrast, Purnell et al (21) reported that secondary surgeries increased with increased length of partnership between partner hospitals and the Smile Train in developing countries. Although, Oginni et al.(24) noted that there was under-reporting of other types of cleft care such as revision surgeries and alveolar bone grafts which are secondary surgeries, the data that was utilised for this present study was comprehensive and captured all the various types of cleft surgeries that were performed during the study period.

Considering that almost ninety percent of our surgeries were primary surgeries the mean age of five years at primary surgeries of this cohort of cleft patients is high. A study from Northern Nigeria reported a mean age of 12.4 yeas in their cohort of 149 patients with cleft anomalies.(25) A Ugandan study(26) reported a mean age of three years while the study by Conway et al. that looked at cleft surgeries across Africa reported a mean age of nine years.(11) A more recent study from south west Nigeria reported a mean age of 2.5 years at primary lip repair.(23) From China an average age of 1.8 years for lip repair and 5.9 years for palatal repair was seen in the study by Kling et al.(12) These contrast with studies from developed countries were timely repair is the norm.(2,27) Reasons for late repair of clefts in developing countries and underserved areas of high income countries are reportedly multifaceted and include low socioeconomic status, lack

of access to care, ignorance and lower educational status of parents of children with clefts.(28,29)

The Millard rotation advancement flap repair was the most employed method of repair for the unilateral cleft lip. This finding was similar to other studies in our sub region and Africa. (20,23,30,31). Although the Von lagenbecks palatoplasty was method more commonly employed for closure of cleft palate the trend showed that tis method had been completely abandoned in the last six years of the study period and replaced with the two flap palatoplasty technique by Bardach. This may probably reflect a change in surgeon dynamics and knowledge oge of palatoplasty techniques as well as the advantages the Bardachs's palatoplasty has over Von Lagenbacks palatoplasty. Von Lagenbeck palatoplsty was however still more commonly used in other similar institutions in Nigeria and amongst cleft surgeons.(13,23,30) The modified Tennison-Randall technique for unilateral cleft lip repair, Bardach's and Von Lagenbeck's methods were favoured in an institution in Italy.(32) The rotation- advancement technique, pushback and double opposing z platies were commoner in Korea.(33) In the United States of America (USA), the Fisher method for unilateral cleft lip repair is gaining popularity over the rotation-advancement techniques. Two-flap palatoplasty with intravelar veloplasty are favoured methods for palatal repair in the USA. (34,35)The reasons why surgeons in Nigeria still stick to the older traditional methods of cleft lip and palate repair needs further exploration.

Prevalence and hazards of late repairs

Cleft surgery of any sort should be individualized based on the objectives of the surgery and the clinical condition of the patient. Nevertheless, various guidelines have been put forward to optimize safety, ensure adequacy of repair, and improve function while limiting morbidity to the

patient with the cleft deformity. Therefore, surgical procedures should be well planned to reduce exposure to anaesthesia from multiple surgeries.(3) One of the common guidelines for the timing of surgical cleft lip repair is the rule of ten.(4) Although its validity is being put to question, it is still widely used.(6–8) This rule is also employed in our institution. The ACPA introduced parameters to evaluate and treat patients with cleft lip and palate.(36) This earlier edition of ACPA guidelines recommended that lip repair be completed by six months and palatal repair by 18 months while the latest revision provides that initiation of lip repair be commenced before 12 months and palatal repair completed by 18 months.(3) They further advised that earlier surgeries are desired if the conditions are right. Early repairs are aimed at improving appearance, speech, hearing, psychosocial development and avoiding impediments to social integration. Based on the ACPA guidelines, Cassell et al. from a resourced nation, reported nearly 90% of the patients in their study had had their surgeries by the age of six months. (27) In this study we found that the prevalence of late primary cleft lip repair was about a third of the patients having primary cleft lip surgery while the prevalence of late palatal repair was more than two thirds of those who received primary palatoplasty. Therefore approximately 60% of those who had cleft lip repair and less than 30% of those who had cleft palate repair met the guidelines as recommended by the ACPA. Despite the availability of free cleft surgeries provided by Smile Train, late repairs are prevalent in our setting.(13,23) interestingly we found that the nature of the deformity was associated with the prevalence of late primary cleft repair. Unilateral cleft lip defects had a higher prevalence of late primary repair than bilateral defects while incomplete clefts of the palate had a higher prevalence of late primary repair than complete defects. Cassell et al. found no association between the type of cleft and timeliness of the repair in their study from North Carolina. (27) We could not find studies that have investigated this relationship in our setting.

The lower prevalence of late primary repair in bilateral defects may reflect the perceived severity of the defects by the parents and the need to seek care to avoid stigmatization. Incomplete defects of the palate are readily recognized, and patients may not present until the speech deficit associated with the deformity becomes apparent. Cassel et al. identified predictors of timely repair to be receipt of maternal care services, access to care and race. Blacks were more likely to have late repairs for orofacial clefts.(27). We also found that amongst children who had cleft lip, incomplete defects had a significantly higher prevalence (44%) of late primary repair when compared with the more severe complete cleft lip and palate (12%). This finding was also mirrored by the palatal defects. Incomplete clefts of the palate had a higher prevalence (68.3%) of late primary repair than complete clefts of the palate (31.8%). Compared with children who had bilateral cleft lip, children with unilateral cleft lip had a significantly increased risk of late primary repair.

Limitations of the study

The data base used for this study was limited in variables such as education of the parent s which may also have affected the late primary repair. The nature of the data base used for this research does not allow for relating the changing trend in the methods of cleft lip and palate repair to outcomes of repair.

Conclusions and Recommendations

The trends in number of cleft surgeries show a low volume in later years. There has ben a change from Von Lagenbeck palatoplasty to Bardachs two-flap palatoplasty. Intra-velar veloplasty and Fisher’s method of lip repair were introduced in later years. There was a higher risk of late

primary repair in children with unilateral cleft lip. There is a need to investigate the reason for the changing trends in techniques of cleft surgeries and determine the impact of this on the functional outcomes of the repairs. More attention would need to be paid to children with less severe cleft deformities to ensure they have early repairs. Increased awareness on identification of incomplete clefts of the palate may increase their recognition and early presentation.

References

1. Vyas T, Gupta P, Kumar S, Gupta R, Gupta T, Singh H. Cleft of lip and palate: A review. Journal of Family Medicine and Primary Care. 2020;9(6):2621.

2. Vanderburg R, Alonso N, Desai P, Donkor P, Mossey P, Stieber E, et al. Age at Primary Cleft Lip Repair: A Potential Bellwether Indicator for Pediatric Surgery. Plastic and Reconstructive Surgery - Global Open. 2021;

3. Parameters for evaluation and treatment of patients with cleft lip/palate or other craniofacial anomalies. American Cleft Palate-Craniofacial Association. Available from: www.acpa.cpf.org accessed September 9 2021.

4. Wilhelmsen HR, Musgrave RH. Complications of cleft lip surgery. Cleft Palate J. 1966;3:223–23.

5. Parameters For Evaluation and Treatment of Patients With Cleft Lip/Palate or Other Craniofacial Differences. The Cleft palate-craniofacial journal : official publication of the American Cleft Palate-Craniofacial Association. 2018 Jan 1;55(1):137–56.

6. Efunkoya AA, Omeje KU, Amole IO, Osunde OD, Akpasa IO. A review of cleft lip and palate management: Experience of a Nigerian Teaching Hospital. Vol. 12, African Journal of Paediatric Surgery. 2015. p. 257–60.

7. Chow I, Purnell CA, Hanwright PJ, Gosain AK. Evaluating the Rule of 10s in Cleft Lip Repair: Do Data Support Dogma? Plastic and Reconstructive Surgery. 2016 Sep 1;138(3):670–9.

8. Acheampong AO, Addison W, Obiri-Yeboah S, Amuasi A, Gowans LJ, Newman-Nartey M, Sabbah DK, Pare P, Selormey R, Baffour G. The Rule Of 10's in the Management of Unilateral Cleft Lip Children: The Komfo Anokye Teaching Hospital Experience. Journal of Clinical Research in Dentistry. 2019;2(1):1-4.

9. Millard DR. A primary camouflage of unilateral harelook. Skoog T, Ivy RH, editors. Baltimore: William and Wilkins; 1957. 163–165.

10. Cassell CH, Daniels J, Meyer RE. Timeliness of primary cleft lip/palate surgery. Cleft Palate-Craniofacial Journal. 2009;46(6):588–97.

11. Conway JC, Taub PJ, Kling R, Oberoi K, Doucette J, Jabs WW. Ten-year experience of more than 35,000 orofacial clefts in Africa. BMC Psychiatry. 2015 Feb 14;15(1).

12. Kling RR, Taub PJ, Ye X, Jabs EW. Oral clefting in China over the last decade: 205,679 patients. Vol. 2, Plastic and Reconstructive Surgery - Global Open. Lippincott Williams and Wilkins; 2014.

13. Onah II, Opara KO, Olaitan PB, Ogbonnaya IS. Cleft lip and palate repair: the experience from two West African sub-regional centres. Journal of Plastic, Reconstructive & Aesthetic Surgery. 2008 Aug 1;61(8):879–82.

14. Smile train arrives Nigeria. Available from: https://nigeriahealthwatch.com/smile-train-arrives-nigeria/

15. Ekwueme AE. Challenges to Optimal Care for Orofacial Cleft Patients in Sub-Saharan Africa - The Example of Two Nigerian Tertiary Hospital. Biomedical Journal of Scientific & Technical Research. 2018 Apr 6;3(4).

16. Oketade I, Bello SA, Adeoye J. The Tale of the Unrepaired Cleft—Retrospective Evaluation of Cases Encountered by an Indigenous Mission in a Northern Nigerian Community. Cleft Palate-Craniofacial Journal. 2021 Jul 1;58(7):888–93.

17. Tessier P. Anatomical classification of facial, cranio-facial and latero-facial clefts. Journal of Maxillofacial Surgery. 1976 Jan 1;4(C):69–92.

18. Orkar KS, Ugwu BT, Momoh JT. Cleft lip and palate: The Jos experience. East African Medical Journal. 2002;79(10).

19. Zhou QJ, Shi B, Shi ZD, Zheng Q, Wang Y. Survey of the patients with cleft lip and palate in China who were funded for surgery by the Smile Train Program from 2000 to 2002. Chinese Medical Journal. 2006;119(20).

20. Olasoji HO, Hassan A, Adeyemo WL. Survey of management of children with cleft lip and palate in teaching and specialist hospitals in Nigeria. Cleft Palate-Craniofacial Journal. 2011;48(2).

21. Purnell CA, McGrath JL, Gosain AK. The role of smile train and the partner hospital model in surgical safety, collaboration, and quality in the developing world. Journal of Craniofacial Surgery. 2015;26(4).

22. Wilson J, Hodges A. Cleft lip and palate surgery carried out by one team in Uganda: Where have all the palates gone? Cleft Palate-Craniofacial Journal. 2012;49(3).

23. James O, Adekunle AA, Adamson OO, Agbogidi OF, Adeyemo WL, Butali A, et al. Management of orofacial cleft in Nigeria - A retrospective study. Annals of Maxillofacial Surgery. 2020;10(2).

24. Oginni FO, Oladele AO, Adenekan AT, Olabanji JK. Original article cleft care in Nigeria: Past, present, and future. Cleft Palate-Craniofacial Journal. 2014;51(2).

25. Akintububo OB, Ojo EO, Kokong DD, Adamu SA, Nnadozie UU, Yunusa-Kaltungo Z, et al. Cleft lip, alveolus and palate in african natives: an update on demographics and management outcome. African journal of medicine and medical sciences. 2014;43(Suppl 1).

26. Hodges S, Wilson J, Hodges A. Plastic and reconstructive surgery in Uganda - 10 Years experience. Vol. 19, Paediatric Anaesthesia. 2009.

27. Cassell C, Daniels J, Meyer R. Timeliness of Primary Cleft Lip/Palate Surgery Among Children with Orofacial Clefts in North Carolina, 1995-2002. The Cleft Palate-Craniofacial Journal. 2009;

28. Adetayo O, Ford R, Martin M. Africa has unique and urgent barriers to cleft care: Lessons from practitioners at the pan-african congress on cleft lip and palate. Pan African Medical Journal. 2012;12(1).

29. Cassell CH, Daniels J, Meyer RE. Timeliness of primary cleft lip/palate surgery. Cleft Palate-Craniofacial Journal. 2009;46(6).

30. Akinmoladun V, Ademola S, Olusanya A. Management of cleft lip and palate in Nigeria: A survey. Vol. 20, Nigerian Journal of Clinical Practice. 2017.

31. Lazarus DD, Hudson DA, van Zyl JE, Fleming AN, Fernandes D. Repair of unilateral cleft lip: A comparison of five techniques. Annals of Plastic Surgery. 1998;41(6).

32. Gatti GL, Freda N, Giacomina A, Montemagni M, Sisti A. Cleft Lip and Palate Repair : Our Experience. J Craniofac Surg. 2017;00(00).

33. Lee TJ, Kim ST. A survey of cleft lip and palate management taught in training programs in Korea. Cleft Palate-Craniofacial Journal. 2003;40(1).

34. Roberts JM, Jacobs A, Morrow B, Hauck R, Samson TD. Current Trends in Unilateral Cleft Lip Care: A 10-Year Update on Practice Patterns. Annals of Plastic Surgery. 2020;84(5).

35. Katzel EB, Basile P, Koltz PF, Marcus JR, Girotto JA. Current surgical practices in cleft care: Cleft palate repair techniques and postoperative care. Plastic and Reconstructive Surgery. 2009;124(3).

36. Parameters for evaluation and treatment of patients with cleft lip/palate or other craniofacial anomalies. American Cleft Palate-Craniofacial Association. The Cleft palate-craniofacial journal. 1993; vol. 30, supplement 1, p. 4.

Acknowledgement

The authors acknowledge Smile Train for funding the surgeries of these patients and provision of the Smile Train Express database. We also acknowledge the National Surgical, Obstetric, Anaesthesia and Nursing Plan (NSOANP)/Smile Train partnership for fostering the collaboration for this work.

Click here to access/download;Figure;Fig 1.tiff

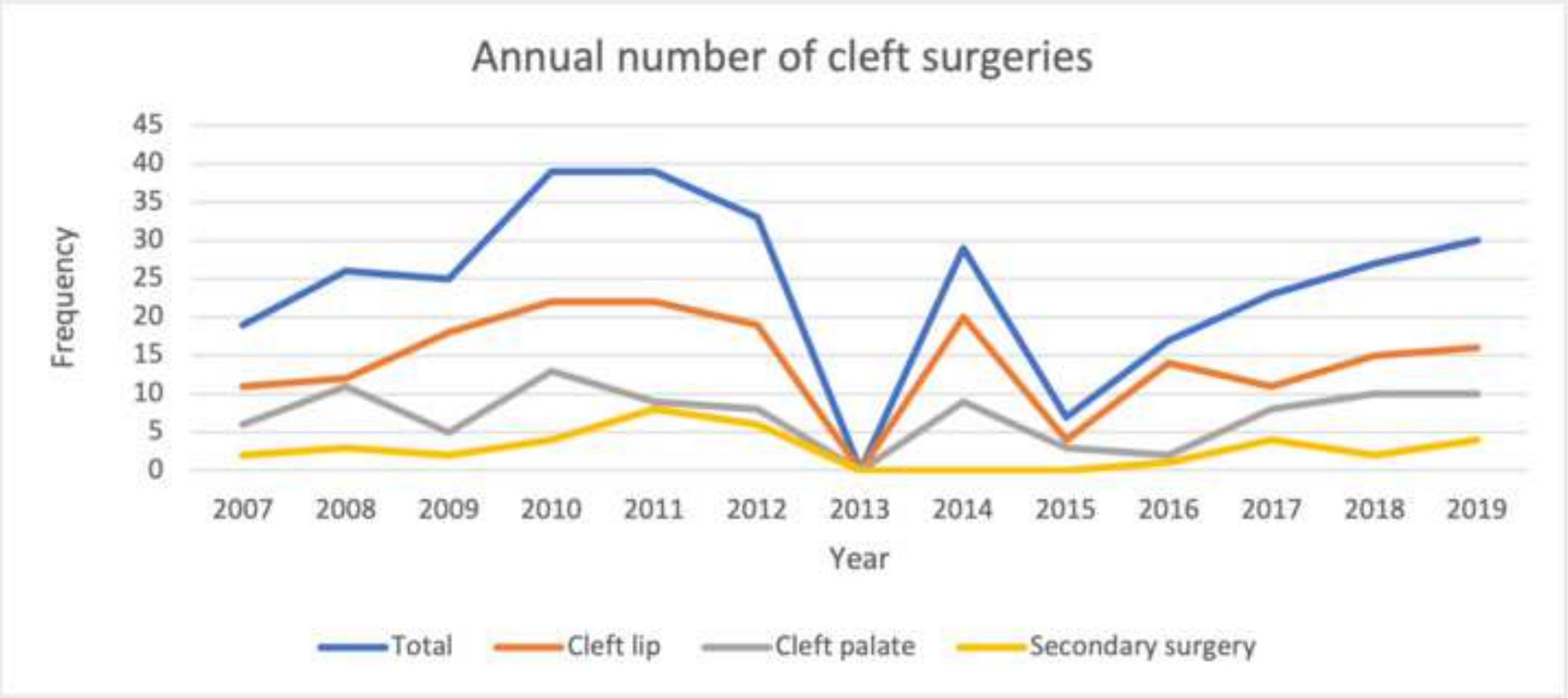

Figure

Click here to access/download;Figure;Fig 2.tiff

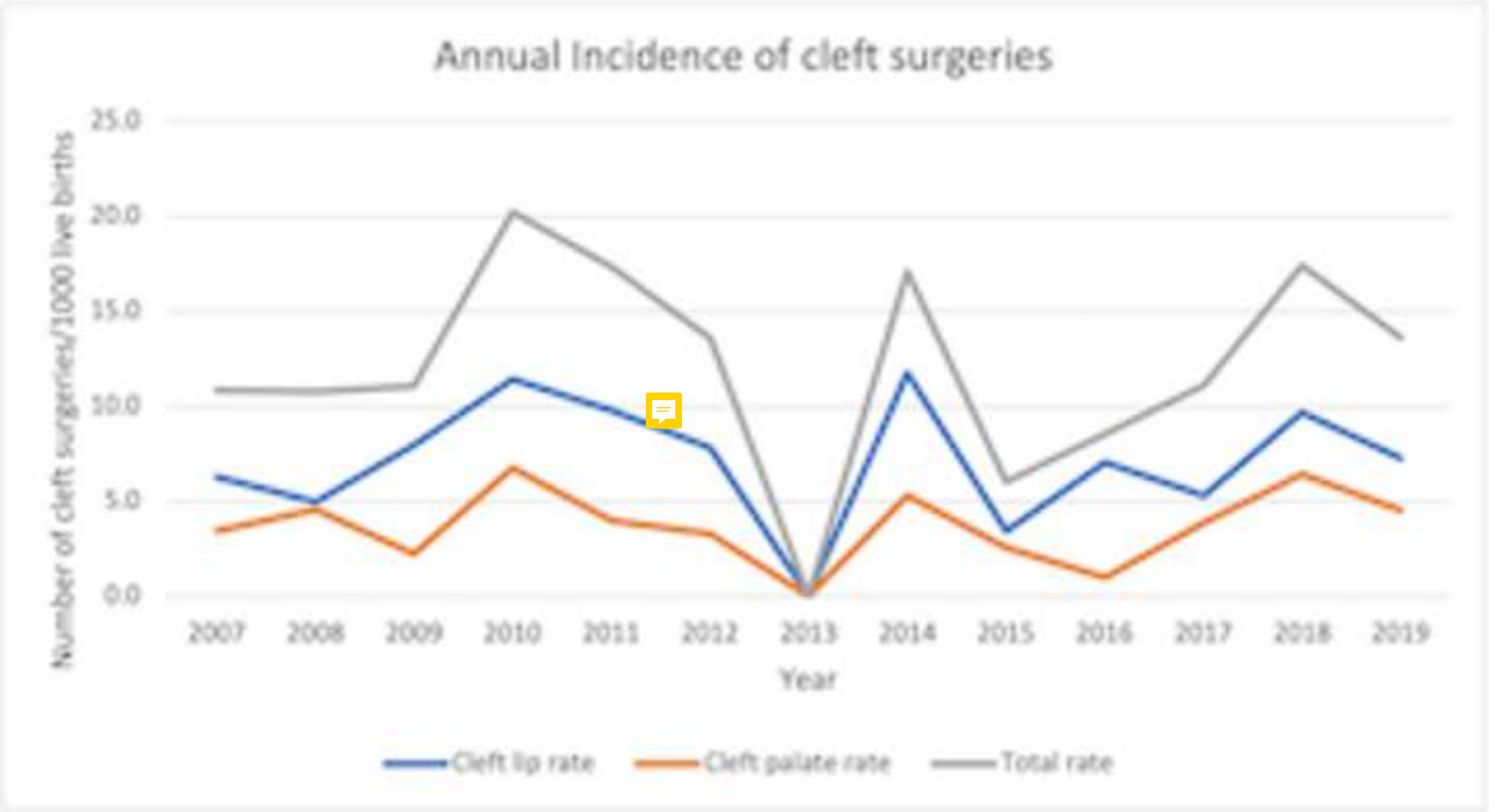

Figure
Click here to access/download;Figure;Fig 3.tiff

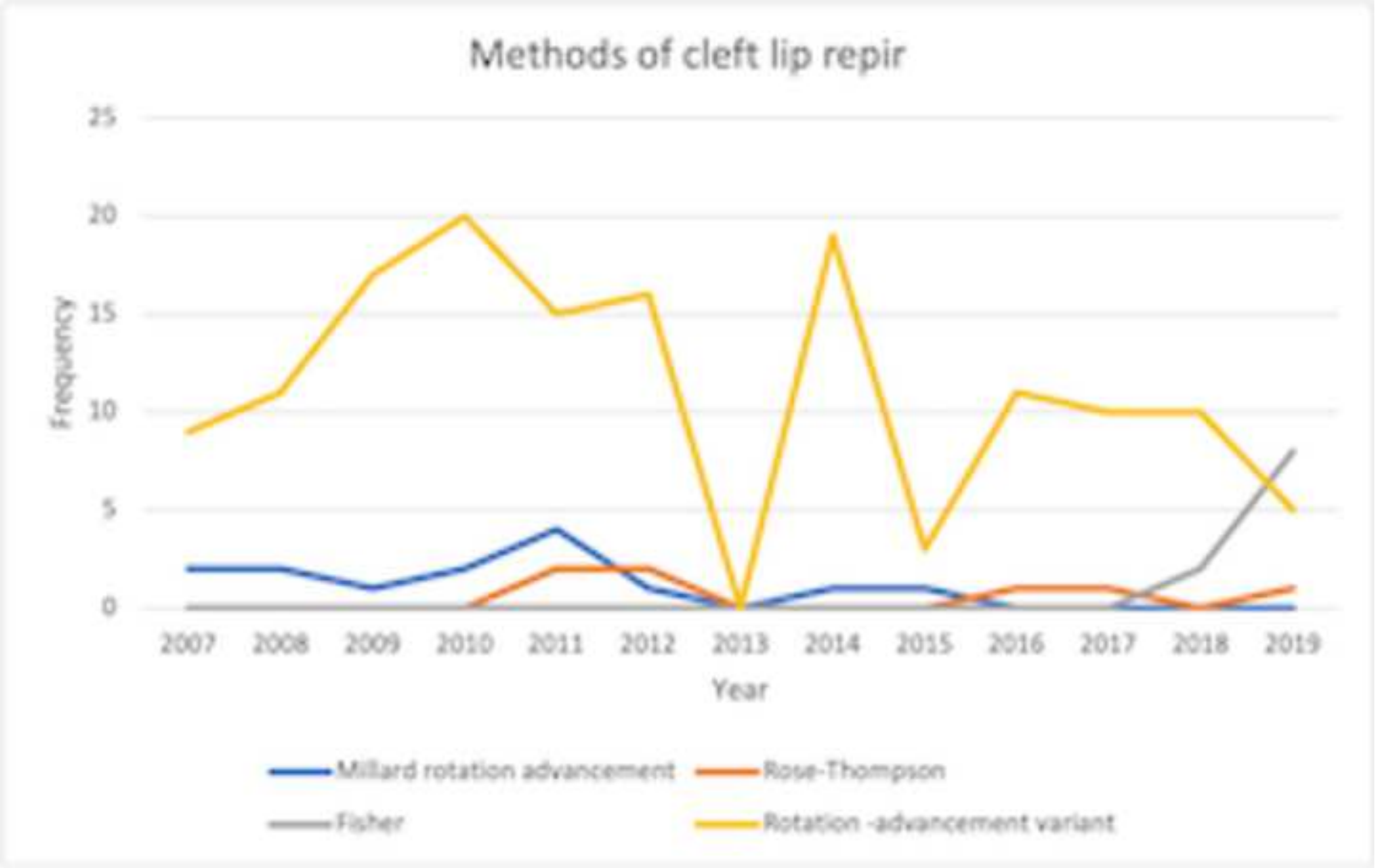

Figure

Click here to access/download;Figure;Fig 4.tiff

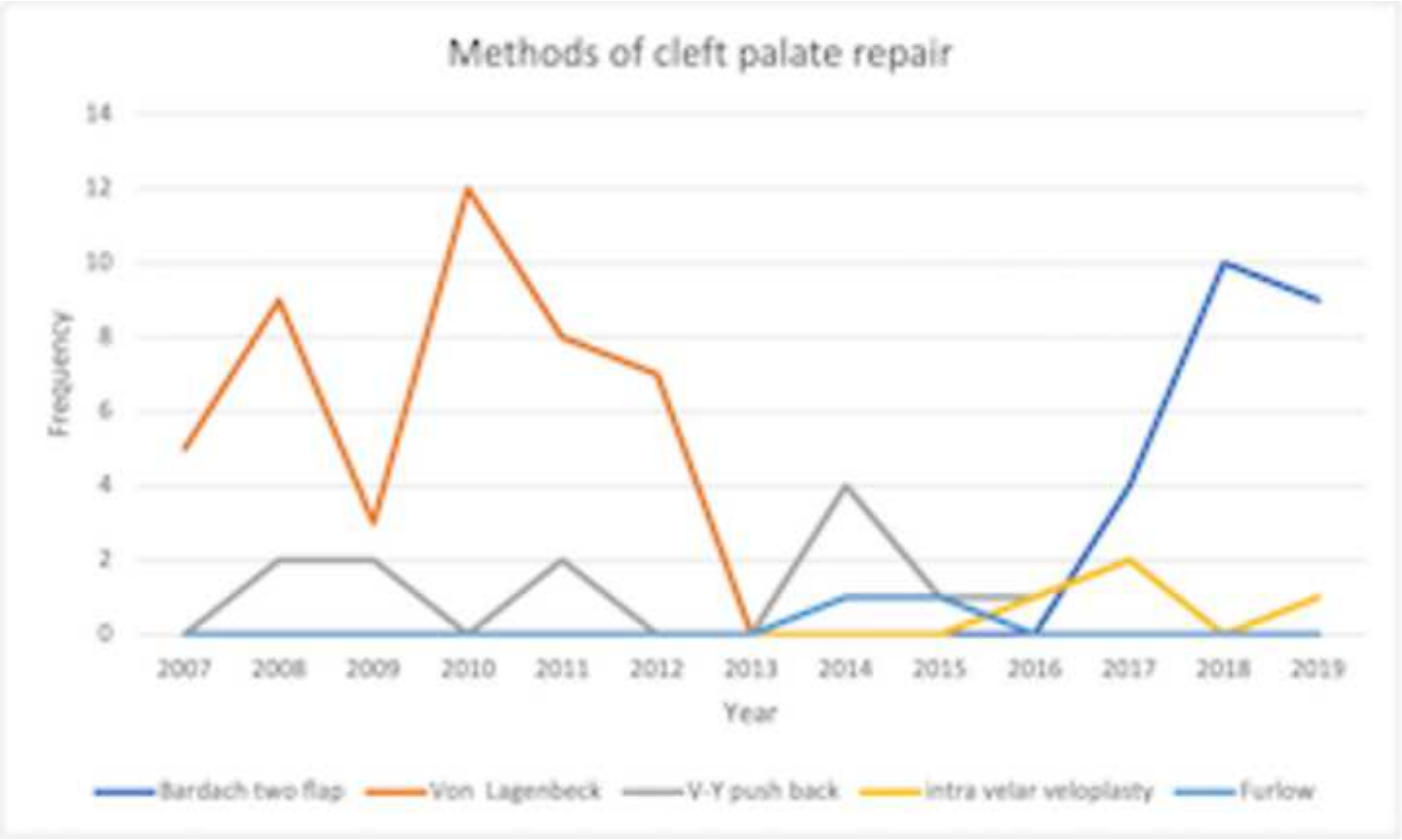

Figure

Click here to access/download;Figure;Fig 5a.tiff

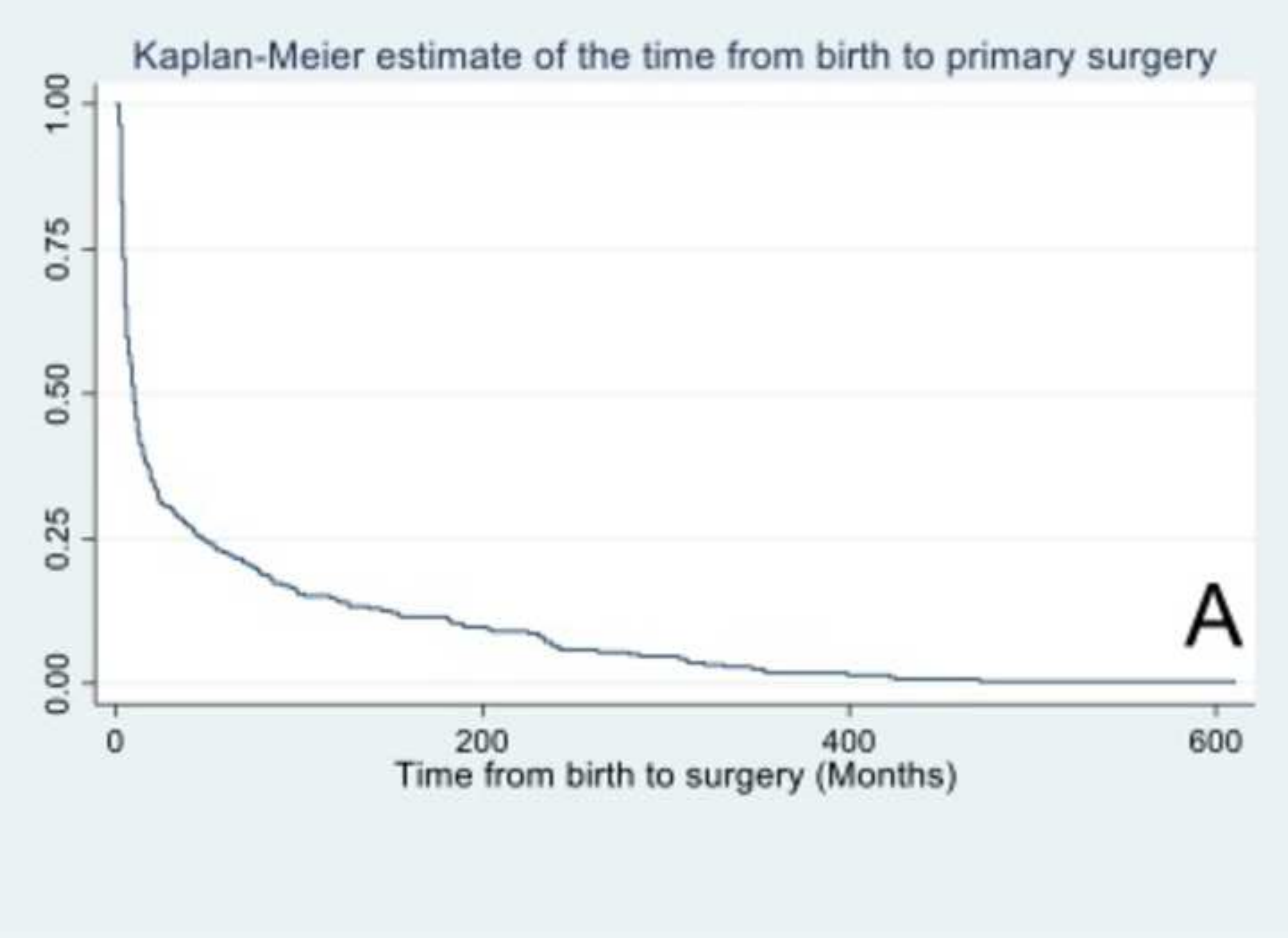

 Click here to access/download;Figure;Fig 5b.tiff

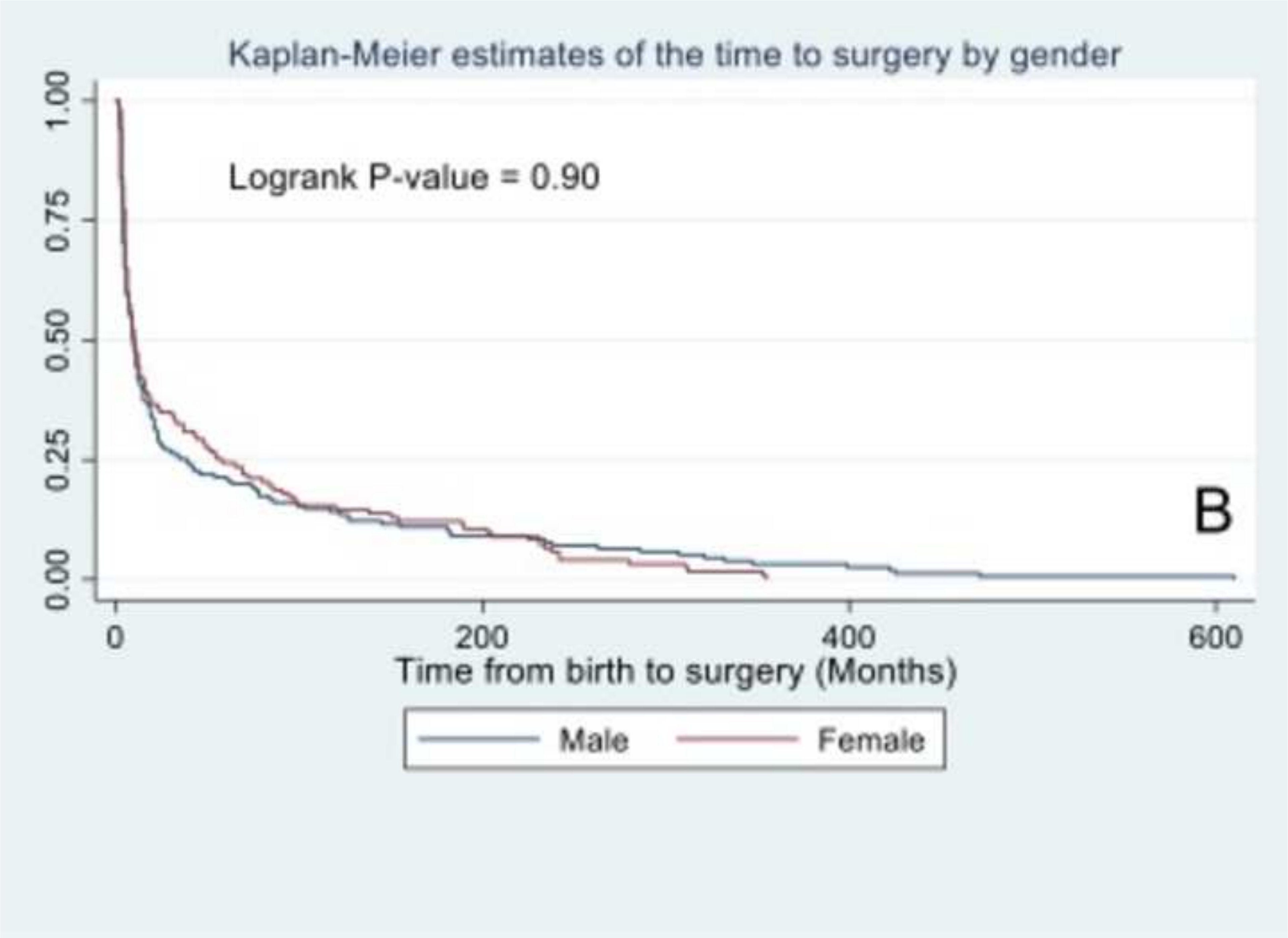

 Click here to access/download;Figure;Fig 5c.tiff

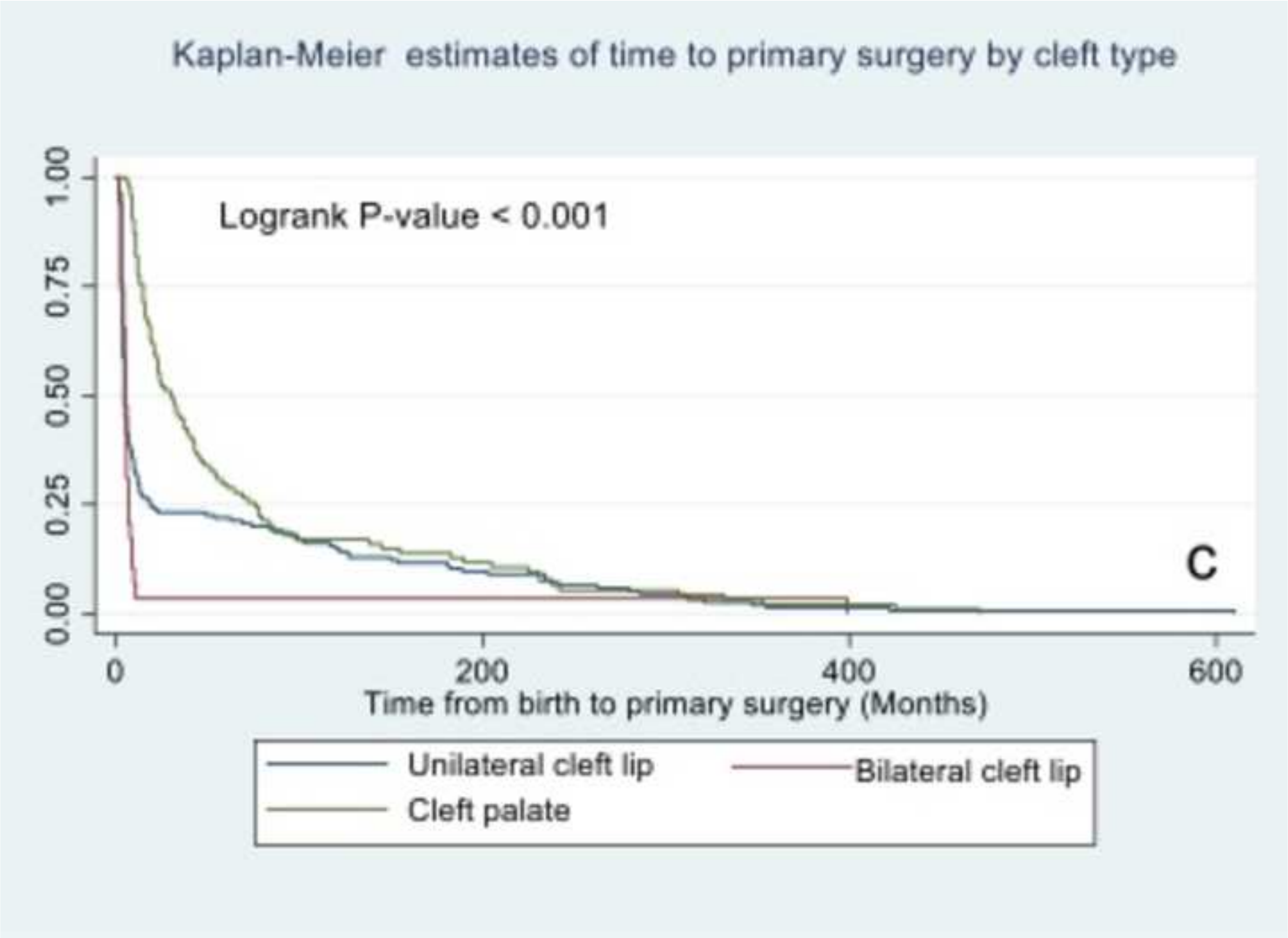

Supplement: Attachment — Submitted filename: PONE-D-22-24340_reviewer01.pdf [file pone.0274657.s001.pdf]
