# Supplementary material for: Trends of cleft surgeries and predictors of late primary surgery among children with cleft lip and palate at the University College Hospital, Nigeria: A retrospective cohort study
Source: PLoS One. 2023 Jan 3;18(1):e0274657. doi: 10.1371/journal.pone.0274657 (PMC9810161; doi:10.1371/journal.pone.0274657)
Supplement: Attachment — Submitted filename: Response to Reviewers comments PLOS ONE.doc [file pone.0274657.s002.doc]

Dear Editor

Plos One

We thank the Academic editor and the reviewers for the review and comments made on our sub mitted manuscript. These comments and corrections have greatly improved our manuscript.

Please find below the response to the comments and queries of the reviewers.

**Reply to academic editor’s comments**

| **Reviewer Number** | **Original comments of the academic editor** | **Response by the authors** | **Location of changes done in manuscript** |
| --- | --- | --- | --- |
| 1 | Please ensure that your manuscript meets PLOS ONE's style requirements, including those for file naming | The manuscript has been formatted to meet PLOS ONE’s style requirements |  |
|  | You indicated that you had ethical approval for your study. In your Methods section, please ensure you have also stated whether you obtained consent from parents or guardians of the minors included in the study or whether the research ethics committee or IRB specifically waived the need for their consent | Due to the retrospective nature of the study the ethics committee waived the need for consent. | Methods  Study design and setting.  Page 7, lines 154,155 159-160 |
|  | Please amend your current ethics statement to address the following concerns: a) Did participants provide their written or verbal informed consent to participate in this study? b) If consent was verbal, please explain i) why written consent was not obtained, ii) how you documented participant consent, and iii) whether the ethics committees/IRB approved this consent procedure. | A written informed consent was obtained from all adult patients and parents of children for surgery, entry of their data into the database and utilization for research. Further need for consent for this study was waived by the ethics committee due to the retrospective nature of the study. | Methods  Study design and setting.  Page 7, lines 154,155 159-160 |
|  | In your Data Availability statement, you have not specified where the minimal data set underlying the results described in your manuscript can be found. PLOS defines a study's minimal data set as the underlying data used to reach the conclusions drawn in the manuscript and any additional data required to replicate the reported study findings in their entirety. All PLOS journals require that the minimal data set be made fully available. | The database that utilized was the database of Smile Train with some restrictions to data availability. Data will be made available on request to the data manager at [stxadmin@smiletrain.org](mailto:stxadmin@smiletrain.org) |  |
|  | Please upload a new copy of Figures 2,3,4,5A,5B and 5C as the detail is not clear. | Clearer figures 2,3,4,5A,5B and 5C have been uploaded | Figures |
|  | Please review your reference list to ensure that it is complete and correct | All the references have been reviewed to comply with PLOS ONE format | References |
| Reviewer 1 | Is there any cleft team was formed at your hospital? as this will offers a comprehensive treatment and follow up to the patients. | Our hospital does not have the full complement of all expected members of a cleft team. The cleft team comprises of cleft surgeons, clinical and public health nurses and a nutritionist. |  |
|  | The hazard of delay in primary repair of cleft are not mentioned in the manuscript although statistical analysis of relationship between the types of cleft and hazard of delay in primary repair was mentioned. What are the hazard involved | We obtained hazard ratio from the Cox proportional hazard regression modelling, and this was reported in Table 5. The hazard referred to is the hazard ratio |  |
|  | Even though authors clearly state the limitation, to absolutely determined the factors that change the surgical approaches, the data related to preoperative assessment, postoperative follow up to compare which surgical approaches causes less complications must be included if the authors really wanted to determine the changing trends of cleft surgical procedures. | We thank the reviewer for this query which is a limitation of the study as stated in the manuscript. The nature of the database used for the study does not allow for this additional information. This study nevertheless has encouraged us to put in a more structured follow up mechanism for a prospective study. |  |
| 2 | For the patients with cleft lip and palate treated at your center, any pre-surgical orthodontic treatment such as Nasoalveolar Molding( NAM) appliances were offered to patients as this will determine surgical approaches that will be provided by the surgeons. | None of the patients had Nasoalveolar moulding. There was no orthodontist on the team within the period of the study |  |
| Spelling errors | Von Lagenbeck | This has been changed to Von Langenbeck | Abstract Subheading Result  Page 3, line 67 |
|  | Von Lagenbeck | This has been changed to Von Langenbeck | Abstract-Conclusion  Page 4, line 79 |
|  | Von Lagenbeck | This has been changed to Von Langenbeck | Results Subheading Trends in methods of cleft lip and palate repair  Page 14, lines 248-249 |
|  | Although the Von lagenbecks palatoplasty was method more commonly employed for closure of cleft palate the trend showed that tis method had been completely abandoned in the last six years of the study period and replaced with the two flap palatoplasty technique by Bardach. This may probably reflect a change in surgeon dynamics and knowledge oge of palatoplasty techniques as well as the advantages the Bardachs’s palatoplasty has over Von Lagenbacks palatoplasty" | *Von Lagenbeck* has been changed to *Von Langenbeck*  *tis* has been changed to *this*.  *Knowlegde oge* has been changed to *knowledge of*. | Discussion  Page 23, line 340  Page 25, line 411  Page 25, line 413 |
| Reviewer 2 | Surgical procedures should be well planned to reduce exposure to anaesthesia from multiple surgeries (3) (One reference is not sufficient to support this statement. Add few more references. The different surgical techniques must also be included) | More references have been added and the different surgical techniques included. | Introduction  Page 5, lines 91-95 |
|  | The procedure of obtaining informed consent is not mentioned. Kindly elaborate the informed consent procedure (Study design and setting) | This had been added to the methods section | Methods  Page 7, lines 154,155 159-160 |
|  | Only primary surgeries of typical clefts were utilized for bivariate and multivariable a analysis to determine the primary outcomes, as such atypical clefts and secondary surgeries were not analyzed further | This sentence has been rewritten as advised | Methods  Page 8, 181-183 |
|  | Further explanation is needed in terms of advancement in latest trends of cleft surgeries. For example, Bardach's technique and other techniques must need a detailed explanation. (Add a new paragraph before Prevalence and hazards of late repairs section. | Additional detail on the surgeries have been added as rightfully advised. These were however embedded into the discussion rather than a separate paragraph in order to maintain the flow of thought/discussion. | Discussion  Page 25, lines 406-409  Pages 25-26, lines 414-475 |
|  | Reference format is not proper. Please refer to ICMJE format for standard journal article | All the references have been revised to comply with Plos One and ICMJE format | References |
|  | The figures must be uploaded in 300 dpi format. (Refer to author guidelines for figures) | The figures have been saved and uploaded in 300dpi format | Figures |

Thank you.

Best regards,

Afieharo Michael

Corresponding author
